# External defect detection of Orah mandarin based on a non-brightness correction algorithm

**DOI:** 10.3389/fpls.2025.1654143

**Published:** 2025-09-12

**Authors:** Panfei Li, Xiaoxiao Jiang, Yuhao Wu, Qiang Fu, Sheng Qin

**Affiliations:** ^1^ Guangxi Key Lab of Brain-Inspired Computing and Intelligent Chips, School of Electronic and Information Engineering, Guangxi Normal University, Guilin, China; ^2^ Key Laboratory of Nonlinear Circuits and Optical Communications (Guangxi Normal University), Education Department of Guangxi Zhuang Autonomous Region, Guilin, China; ^3^ College of Physical Education and Health, Guangxi Normal University, Guilin, China

**Keywords:** defect detection, non-brightness correction, sliding window, histogram statistics, morphological operations

## Abstract

External defect detection is a crucial step in Orah mandarin citrus grading. However, in existing defect detection algorithms by image processing, Orah mandarin surfaces exhibit characteristics such as higher brightness at the center, lower brightness at the edges, and uneven brightness distribution in images. Although traditional brightness correction algorithms can solve these issues, they suffer from limitations including prolonged processing time, high computational complexity, and elevated false detection rates. To address these shortcomings, this work proposes a non-brightness correction algorithm to enhance the speed and accuracy of Orah mandarin external defect detection. The proposed algorithm divides Orah mandarin images into multiple equal-sized regions and performs threshold segmentation sequentially using a sliding window matching the region size. A sliding window size of 100 × 100 pixels was chosen because it offers a balanced trade-off between detection precision and computational efficiency, allowing the algorithm to detect both large and subtle defects effectively while maintaining fast processing speed. First, the histogram statistical method categorizes the current sliding window region into three types, and a dedicated defect detection algorithm applies adaptive thresholding to each type. Next, the threshold-segmented regions are merged, while the fruit stem area is excluded by combining circularity and hue features. Finally, morphological operations eliminate noise to obtain complete defect segmentation results. Experimental results demonstrate that with a sliding window size of 100 × 100 pixels, the algorithm achieves rapid external defect detection at 85.3 ms per fruit and a 97.5% defect recognition rate, offering a novel approach for fruit surface defect detection. This performance is consistent across different defect types, though the algorithm performed best for point-like defects, such as thrips scarring and canker spots, where clear, localized defects were more easily detected. For blocky rot defects, such as sunburn, the algorithm exhibited a slightly lower recognition rate, particularly in areas where the defect was less distinct and more integrated with the fruit’s surface. These findings suggest that the algorithm is effective for a range of defect types but may require further refinement to handle more complex or overlapping defects.

## 1 Introduction

Orah mandarin exhibit a quasi-spherical shape, resulting in lower grayscale values at image edges compared to the central regions even under soft illumination conditions (Castro et al., 2023). This characteristic frequently leads to misidentification of edge areas as defects during detection, necessitating brightness correction algorithms for image preprocessing to enhance external defect detection accuracy. Brightness correction algorithms are commonly employed to eliminate uneven brightness or illumination variations in images (Zhang et al., 2020; He et al., 2022; Cao et al., 2018), aiming to achieve uniform brightness and contrast across the image. Widely used methods include histogram equalization (Pizer et al., 1987; Patel and Goswami, 2014), hyperbolic tangent transformation (Yan, 1996), Retinex theory-based algorithms (Land and McCann, 1971), and illumination-reflectance theory-based algorithms (Shang et al., 2023). Histogram equalization enhances defect visibility by redistributing pixel intensities to broaden the brightness range (Abdullah-Al-Wadud et al., 2007). However, it often introduces over-enhancement, noise amplification, and artifacts while compromising image details. The hyperbolic tangent transformation nonlinearly maps grayscale values to the [-1, 1] range for uniform distribution, primarily optimizing global contrast at the expense of local detail distortion or blurring. Retinex theory-based algorithms mimic human visual perception by separating low-frequency illumination and high-frequency reflectance components (Bertalmío et al., 2009), effectively addressing uneven illumination but suffering from high computational complexity and slow processing speeds for large-scale datasets. Illumination-reflectance decomposition methods enhance dynamic range by decoupling image brightness (illumination) and texture details (reflectance), yet they demand substantial computational resources and prolonged processing time. While global brightness correction algorithms partially mitigate uneven brightness distribution, they often compromise local details, increasing false defect detection rates and computational overhead. Non-brightness correction approaches avoid such preprocessing, instead adopting region-oriented segmentation strategies. These methods partition target images into subregions with homogeneous features, preserving intra-region pixel consistency to facilitate subsequent analysis. When sufficiently small, these subregions exhibit nearly uniform brightness due to inherent pixel similarity and connectivity.

This study first reviews existing non-brightness correction fruit defect detection methods. An improved sliding window-based local segmentation algorithm is then developed, incorporating three contributions: 1) classification-based processing of sliding window regions, 2) optimized window traversal frequency and segmentation areas, and 3) parameter optimization through multi-scale window testing. Systematic experiments with varying window sizes and inter-class grayscale difference thresholds enable rapid and effective Orah mandarin surface defect detection without brightness correction, achieving balanced performance in speed and accuracy.

The rest of the paper are organized as follows: Section 2 presents related works. Section 3 provides a detailed description of the proposed method. Section 4 reports the experimental results and experimental analysis. Finally, a conclusion is presented in Section 5.

## 2 Related works

Traditional image processing approaches have strong interpretability, enabling intuitive visualization of defect information. These approaches typically require no extensive training data or complex model, ensuring fast processing speeds suitable for real-time applications or time-sensitive scenarios. Region-oriented segmentation algorithms partition images into bounded subregions for localized feature extraction, outperforming global segmentation methods in targeted area delineation and defect detection precision.

The region growing method, a widely used region-based segmentation technique, clusters pixels into homogeneous subregions and has been extensively applied in fruit defect detection (Yang and Marchant, 1996; Yogesh et al., 2020; Peng et al., 2021). For instance, Blasco et al. (2007) achieved 95% accuracy in citrus defect detection by forming uniform color regions through region growing and subsequent noise removal. However, their method failed to distinguish stems from defects.

In contrast, sliding comparison window algorithms excel in localizing subtle defects with higher precision. Rong et al. (2017a) proposed a local segmentation method using adaptive thresholds to classify windows as defective or normal. While effective for high-contrast defects, it underperformed on defects with grayscale similarities to healthy areas (e.g., thrips-damaged or sunburned fruits). Their reliance on aspect ratios for stem identification also caused misclassification of defect-like features. Moallem et al. (2017) combined morphological operations with a Mahalanob is distance classifier for apple stem detection, but encountered false positives when stems resembled defects in color and position. To address this issue, Yang et al. (2023a) proposed a defect detection method based on image segmentation and stem/calyx recognition (Pham and Lee, 2015). adopted K-means clustering with Euclidean distance for segmentation, followed by noise filtering and minimum spanning tree-based merging. Though outperforming Otsu’s method in segmentation quality, their algorithm incurred high computational costs. For speed optimization (Soltani Firouz and Sardari, 2022), combined machine vision and image processing technologies, using a special camera to capture images of agricultural products, evaluating the images through image processing techniques, and then using classification algorithms to classify the products based on detected defects. This method has a simple structure and fast calculation speed, but due to uneven surface distribution of fruits, there are still shortcomings in error detection. To solve this problem, Rong et al. (2017b) proposed a simple and easy to implement fast adaptive brightness correction algorithm, which significantly improved the recognition accuracy of most defects on the surface of fruits. However, the recognition effect of defects in the edge area of fruits still needs to be improved.

Recent works have introduced advanced techniques that enhance defect detection performance. For example, ASFM-AFD (Li et al., 2025) presents a multimodal fusion approach that integrates LiDAR and camera data, improving defect detection accuracy by combining complementary information from different sensors. This fusion method has been shown to outperform single-modality approaches, particularly in challenging defect detection scenarios where one modality may fail to capture critical features. However, the method’s reliance on specialized hardware increases its complexity and computational requirements, making it less suitable for real-time applications compared to simpler, image-only methods like the one proposed in this study.

Additionally, the hybrid YOLO model with an attention mechanism (Li et al., 2024) offers significant improvements in defect detection by enhancing the model’s focus on important areas of the image. This attention mechanism allows the network to concentrate on subtle defects, leading to higher precision, especially for small or occluded defects. While this approach offers superior performance in terms of accuracy, it requires substantial computational resources for training and inference, which may limit its applicability in resource-constrained environments.

Meanwhile, deep learning techniques, particularly those combining methods like Fourier decomposition and deep learning models(X. Li, Lin, et al., 2025), have been proven to enhance the interpretation and accuracy of complex data. By utilizing Fourier decomposition’s frequency-domain properties alongside deep learning’s powerful feature extraction capabilities, these methods can significantly improve detection accuracy, particularly in domains with complex textures or varying illumination. This approach shows promise not only in medical or signal processing fields but also for defect detection in images with challenging conditions. Future research could explore integrating such hybrid methods for more These advancements highlight the trade-offs between computational complexity and detection accuracy. While these state-of-the-art methods offer improved performance, they often come at the cost of higher computational demands, making simpler and more efficient methods, like the one proposed in this study, more practical for real-time defect detection in certain applications. robust image-based defect detection.

## 3 Non-brightness correction Orah mandarin defect detection algorithm

### 3.1 Background segmentation algorithm

Background segmentation of Orah mandarin images refers to separating the foreground (Orah mandarin) from the background, thereby enhancing the prominence of the target object for subsequent processing (Chacon-Murguia et al., 2015). This critical preprocessing step improves the accuracy and efficiency of downstream tasks such as object detection and tracking. The algorithm in this work operates as follows: the first is Color Space Conversion—Convert the Orah mandarin image to the HSV (Hue, Saturation, Value) color space (Sural and Pramanik, 2002); the second goes to S-Channel Thresholding—Apply Otsu’s thresholding method (Otsu et al., 1975) to the Saturation (S) component to generate an initial binary mask; the third falls to Morphological Refinement—Perform morphological opening and closing operations to eliminate burrs and fill holes in the threshold-segmented image, and the fourth is Background Removal—Use the refined binary mask to execute a bitwise AND operation with the original image, effectively isolating the Orah mandarin fruit by removing the background. This pipeline ensures robust background elimination while preserving critical foreground details, laying the foundation for subsequent defect detection stages.

To improve computational efficiency, the S-channel extraction and Otsu threshold calculation steps are performed only once at the beginning of the process. The results from these steps are then reused for both the background segmentation and sliding window thresholding stages, thus eliminating redundant computations and improving overall processing speed without sacrificing detection accuracy.

### 3.2 Sliding window threshold segmentation algorithm

The sliding window threshold segmentation algorithm is a local thresholding-based image segmentation method (Pappas and Jayant, 1989). This algorithm first divides an image into multiple sub-regions and then selects an appropriate threshold within each region to perform binarization, thereby separating the image into foreground and background. Specifically, the algorithm scans the target image using a sliding rectangular window, computes an optimal local threshold for each window, and applies this threshold to binarize pixels within the window. Common local threshold calculation methods include Otsu’s method and Sauvola’s algorithm (Sauvola and Pietikak, 2000). By allowing adaptive thresholds across different regions, this approach effectively handles variations in image characteristics, such as uneven illumination or complex backgrounds.

To address the uneven brightness distribution on Orah mandarin surfaces, the acquired Orah mandarin image $I_{\text{orange}}$ is uniformly divided into *N* sub-blocks. Theoretically, when *N* is sufficiently large, the brightness within each sub-block can be approximated as uniformly distributed. However, due to inherent correlations between adjacent sub-blocks, a secondary partitioning is applied to the Orah mandarin image to enhance defect detection accuracy.

With the goal of enhancing defect detection performance, we conducted a sensitivity analysis to evaluate the effect of varying sliding window sizes and circularity thresholds on both detection accuracy and computational efficiency. The sensitivity analysis revealed that smaller sliding windows led to higher detection accuracy, but at the cost of increased computation time. On the other hand, larger windows enhanced processing speed but resulted in the loss of smaller defects. We also observed that the choice of circularity threshold significantly impacted detection performance, especially in distinguishing defects from non-defective regions. Based on our tests, we determined that a circularity threshold of 0.8 provided the optimal balance between minimizing false positives and ensuring high detection accuracy. Let the target Orah mandarin image be denoted as $I_{\text{orange}}$ , with its pixel matrix represented by Equation (1).


(1) $$I_{orange}=[\begin{array}{cccc} a_{11} & a_{12} & \cdots & a_{1y}\\ a_{21} & a_{22} & \cdots & a_{2y}\\ \cdots & \cdots & \cdots & \cdots \\ a_{x1} & a_{x2} & \cdots & a_{xy} \end{array}],$$

where $a_{ij}\;$ ( i=1,2,…,x; j=1,2,…,y ) represents the pixel value at the corresponding position in the image, and the size of $I_{\text{orange}}$ is x×y . The partitioned image of $I_{\text{orange}}$ is denoted as matrix $I_{\text{orange}2}$ , as shown in Equation (2).


(2) $$I_{orange2}=[\begin{array}{cccc} I_{11} & I_{12} & \cdots & I_{1n}\\ I_{21} & I_{22} & \cdots & I_{2n}\\ \cdots & \cdots & \cdots & \cdots \\ I_{m1} & I_{m2} & \cdots & I_{mn} \end{array}],$$

where $I_{ij}\;$ ( i=1,2,…,m; j=1,2,…,n ) represents an image matrix of size p×q . Each $I_{ij}$ corresponds to a sub-block image matrix obtained by uniformly dividing $I_{\text{orange}}$ into *N* windows. The sub-block image matrix $I_{ij}$ is expressed by Equation (3).


(3) $$I_{ij}=[\begin{array}{cccc} a_{11} & a_{12} & \cdots & a_{1p}\\ a_{21} & a_{22} & \cdots & a_{2q}\\ \cdots & \cdots & \cdots & \cdots \\ a_{p1} & a_{p2} & \cdots & a_{pq} \end{array}].$$

**Figure 1:**
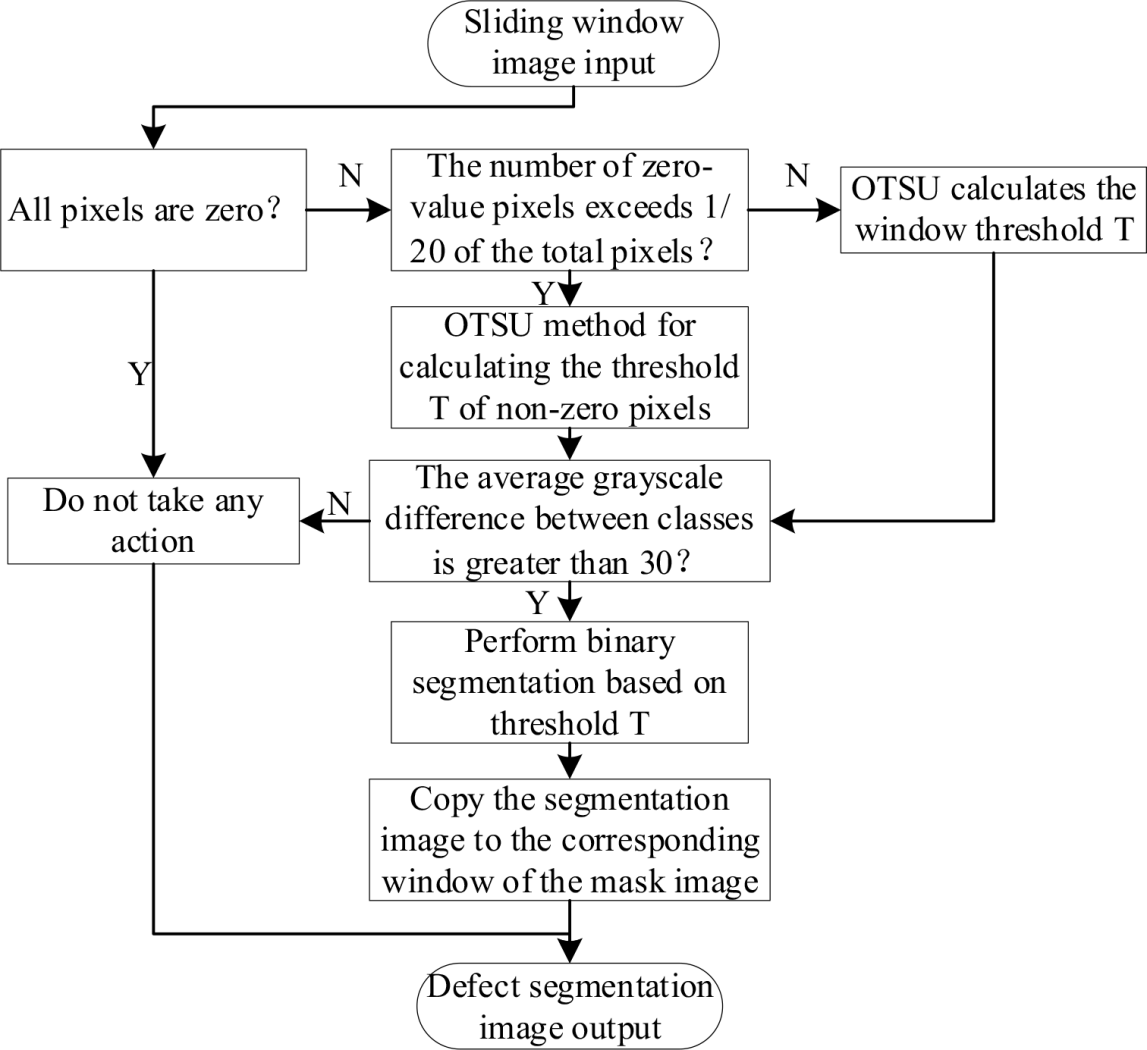
Sliding window threshold segmentation algorithm flow.

The dimensions of $I_{ij}$ are calculated as: p=x/n and q=y/n . This work introduces refinements to the calculation method for the optimal threshold *T* in the non-brightness correction algorithm (Rong et al., 2017b), designing the algorithmic workflow as illustrated in **Figure 1**. The algorithm categorizes sliding window sub-blocks into three types; they are Background blocks—Sub-blocks where all pixels are zero, representing non-target “background” regions; Edge blocks—Sub-blocks where the number of zero-value pixels exceeds 1/20 of the total pixels, indicating Orah mandarin edge regions, and Foreground blocks: All other sub-blocks, representing the central “foreground” regions of the Orah mandarin. Distinct algorithms tailored to each block type are applied to process window image data, enhancing adaptability to regional characteristics.

**Figure 2:**
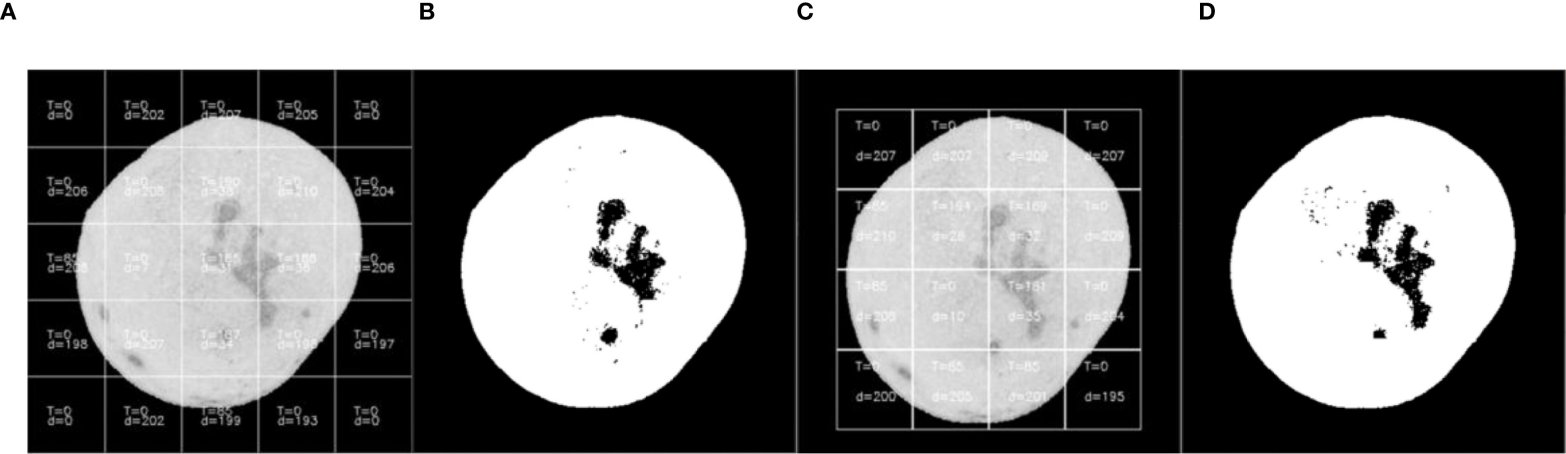
The result of threshold segmentation based on maximum inter-class variance method. **(A)** First segmentation with parameter annotations, **(B)** Binarization result of first segmentation, **(C)** Second segmentation, **(D)** Binarization result of second segmentation.

**Figure 3:**
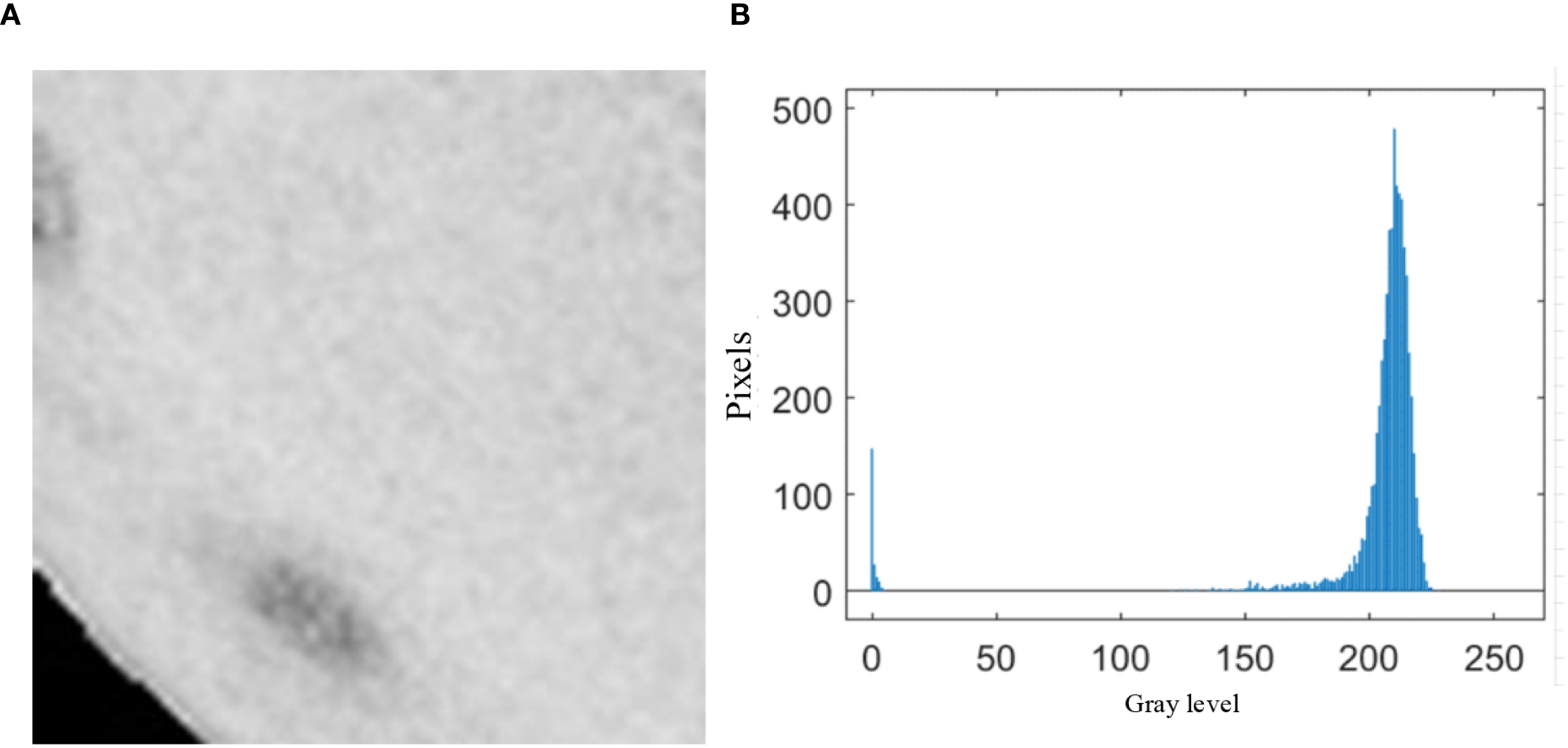
Analysis of sliding window histogram.

**Figure 4:**
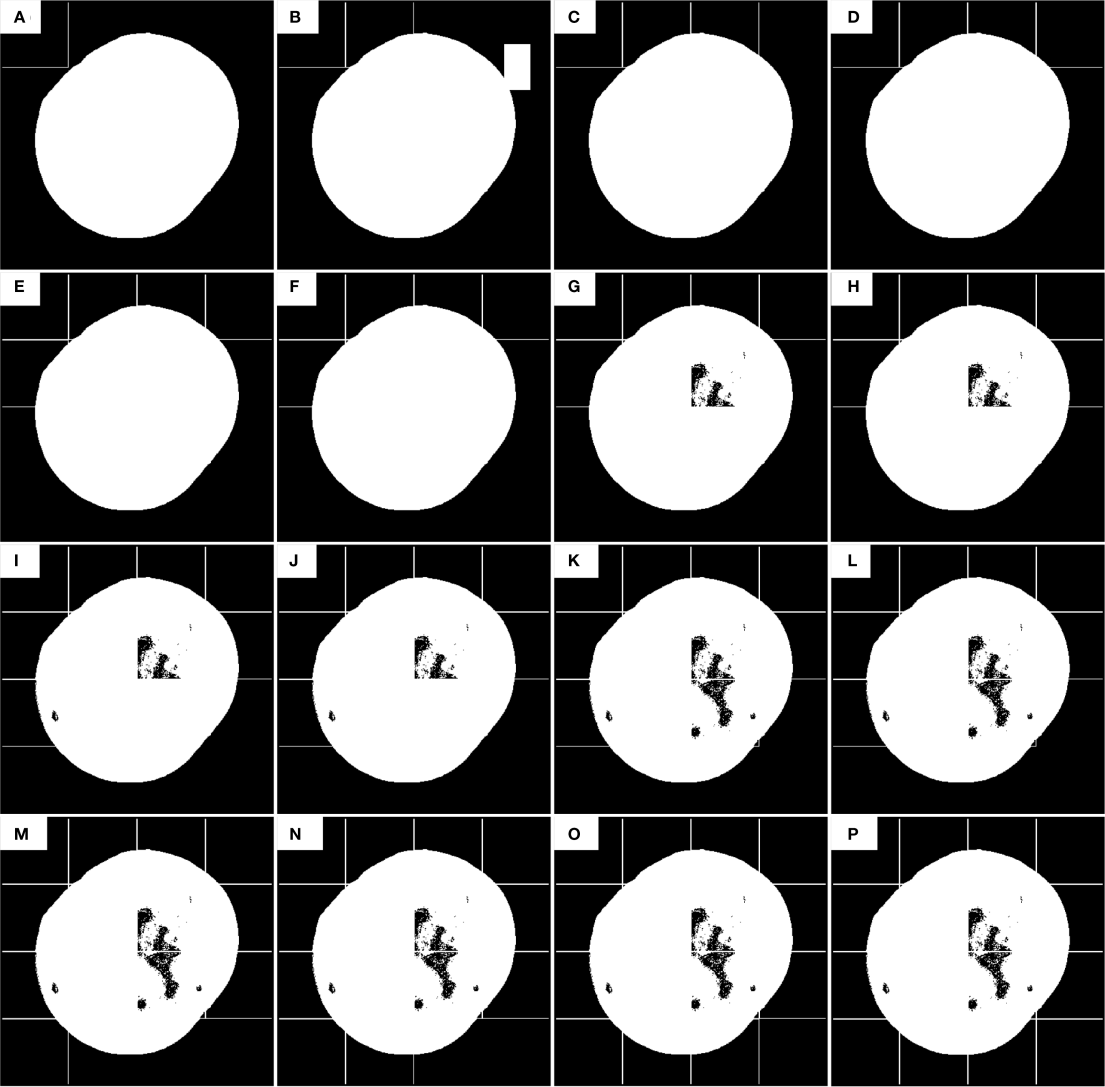
The first defect detection process of the improved sliding window algorithm. **(A–P)** is the position detected by the current sliding window.

**Figure 5:**
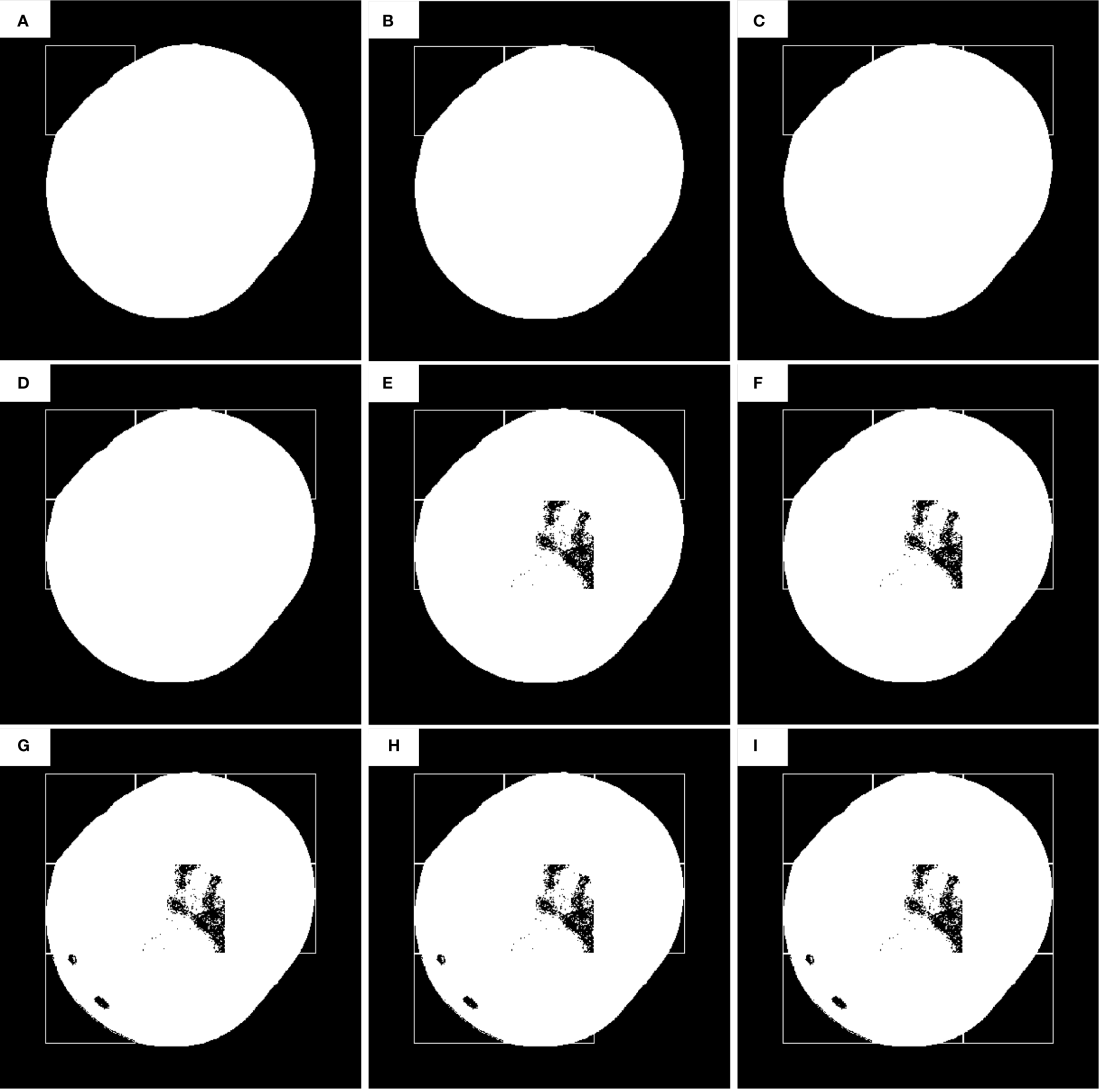
The second defect detection process of improved sliding window algorithm. **(A–I)** is the position detected by the current sliding window. **(A)** Offset initial position (x₀=n/2, y₀=n/2), **(B–I)** Sliding steps (d_step_=n).


The optimized algorithm effectively meets the requirements for Orah mandarin surface defect detection. The detailed algorithmic steps are as follows:

1) Image Preprocessing: Crop the acquired Orah mandarin image to 400 × 400 pixels and remove the background to obtain the target detection image $I_{orange}$ .
2) Sliding Window Configuration: Define a sliding window $I_{w}$ of size n×n , with the relationship between window size n and traversal steps *N* (to fully scan the image) is given by Equation (4).

(4) n×n=400×400÷N.

3) Mask Generation: Extract the mask image $I_{\text{mask}}$ from the background segmentation step for subsequent binarization.
4) S-Channel Extraction: the Saturation (S) channel from $I_{\text{orange}}$ to create the defect segmentation target image $I_{S}$ .
5) Primary Window Partitioning: Initiate scanning from the top-left corner of $I_{S}$ . Move the sliding window $I_{w}$ by *n* pixels after each detection cycle to achieve row-by-row and column-by-column traversal.
6) Pixel Analysis & Segmentation: Calculate pixel statistics within $I_{w}$ ‘s coverage area. Perform detection using the sliding window segmentation workflow outlined in **Figures 2**–**5**. The inter-class average grayscale difference is calculated as shown in Equations (5–7).

(5) $$m_{1}(k)=\sum_{i=1}^{k}iP(\frac{i}{C_{1}}),$$

(6) $$m_{2}(k)=\sum_{i=k+1}^{L}iP(\frac{i}{C_{2}}),$$

(7) $$\Delta d=|m_{1}(k)-m_{2}(k)|,$$

where $C_{1}$ represents Pixels with gray levels (1,2,…,k) , *k* is the optimal segmentation threshold T determined by Otsu’s method. $C_{2}$ represents Pixels with gray levels (k+1,k+2,…,L) , *L* is the maximum gray level within the window. p(i) represents the frequency distribution of gray level *i*. m(k) is the average gray value for levels 1 to *k*. Δd is the inter-class average grayscale difference within the window.

7) Secondary Window Partitioning: Apply a positional offset equal to half the primary window size, i.e., initial coordinates $x_{0}=n/2$ , $y_{0}=n/2$ . Set the sliding step length *d* step equal to the window size *n*, i.e., $d_{step}=n$ .
8) Image Fusion: The final defect-containing binary image ${\text{I}}_{\text{b}}$ is obtained by a weighted combination of the two binarized masks as shown in Equation (8).

(8) $$I_{b}=\alpha \cdot I_{b1}+\beta \cdot I_{b2},$$

where ${\text{I}}_{\text{b}1}$ and $I_{b2}$ are the binarized masks from the first and second sliding−window passes, respectively, and α and β are their weights ( α>β≥0 ). 9) Morphological Refinement: Perform morphological operations to eliminate noise, yielding the final refined defect segmentation binary image.

### 3.3 Stem detection algorithm

Stem detection aimed at improving Orah mandarin defect detection accuracy. In surface defect detection, stems often differ from normal fruit surfaces and may be misclassified as defects. Additionally, during external quality assessment, the color similarity between stems and immature Orah mandarin surfaces can compromise maturity evaluation accuracy (Li et al., 2015). Thus, eliminating stem interference is essential. This work employs color feature extraction and morphological analysis to identify stems. When a defect segmented by the sliding window algorithm aligns with the hue distribution (Li et al., 2022) and morphological characteristics of stems, it is classified as a stem and annotated. Key features include Morphological criterion: Circularity of the defect region (Seng and Mirisaee, 2009), and Color criterion: Average H hue value of the defect region (Yang et al., 2023b).

## 4 Experimental results and analysis

This section presents the details of the dataset and experimental setup, and provides experimental results and performance analysis.

### 4.1 Experimental dataset and platform

**Figure 6:**
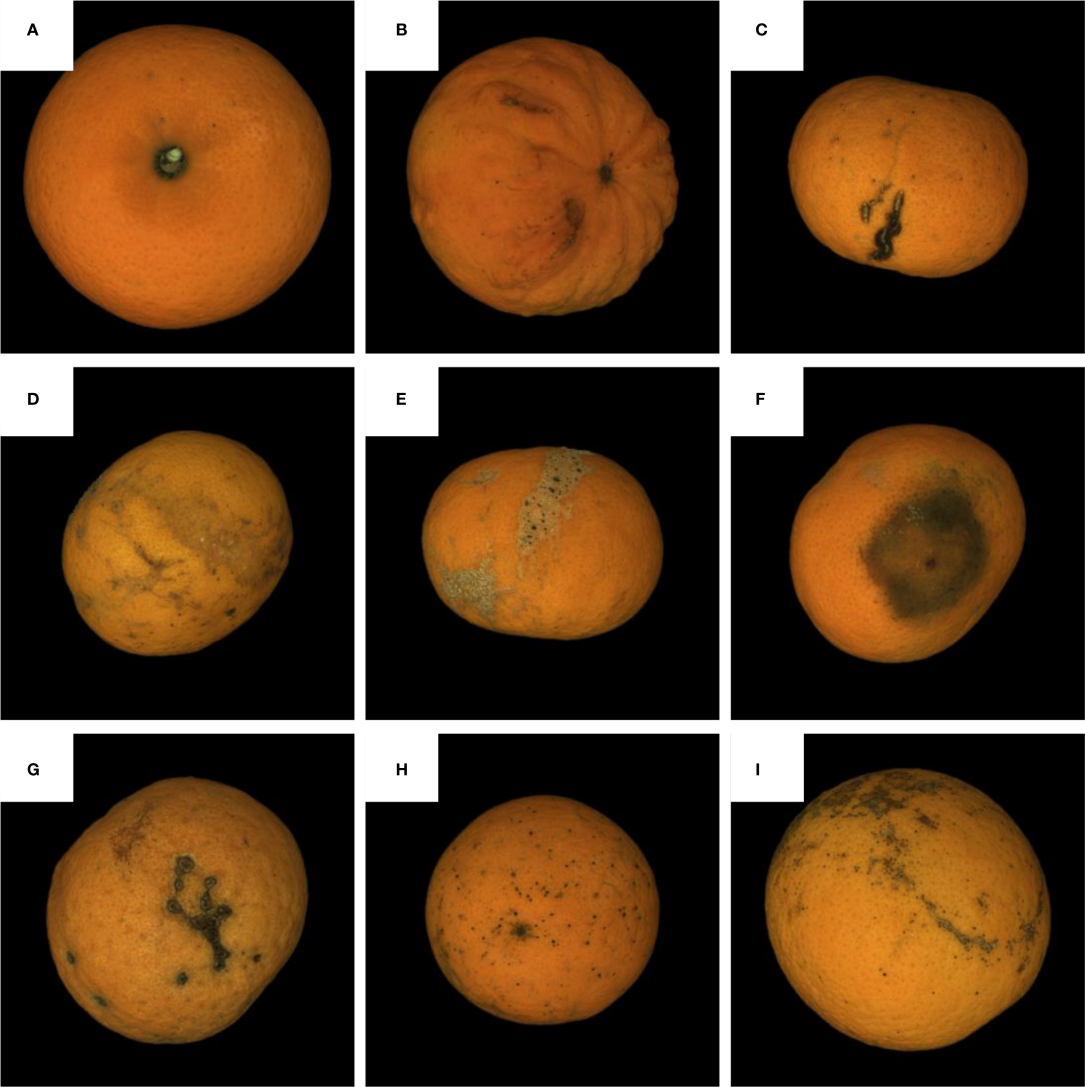
The samples of Orah mandarin dataset. **(A)** The sound Orah mandarin, **(B)** rough pear, **(C)** Coarse skinned fruit, **(D)** The thrips scarring, **(E)** white scaled lesion, **(F)** Phyllocoptruta oleivora, **(G)** The canker spot, **(H)** Citrus scab and **(I)** The copper burn.

This study employs Guangxi Orah mandarin as research subjects. Among the 100 collected samples, the dataset includes normal Orah mandarin specimens and specimens with common diseases/pests (canker, gray mold, thrips, rust mites, scab, white rot, sunburned fruits, and rough peel). The experimental results reported throughout this study are derived from the average of 100 images, as shown in **Table 1**, ensuring that the results are robust and representative of the target population. The samples of data as shown in **Figure 6**. The algorithm was developed and validated based on VS2019 and OpenCV, implemented on a Windows-based host machine (Hardware configuration: Intel(R) Core(TM) i5–9500 CPU @ 3.3GHz, 24GB RAM, etc.).

**Table 1:** Dataset distribution of Orah mandarin samples.

Sample type	Defect type	Quantity	Proportion (%)
Normal	Sound fruit	20	20.0
Rough peel	Surface irregularity	12	12.0
Coarse skin	Texture anomaly	10	10.0
Thrips scarring	Insect damage	15	15.0
White rot	Fungal infection	8	8.0
Citrus rust mite	Pest damage	13	13.0
Citrus canker	Bacterial spot	11	11.0
Citrus scab	Fungal scab	6	6.0
Sunburn	Environmental damage	5	5.0
Total		100	100

### 4.2 Background segmentation

**Figure 7:**
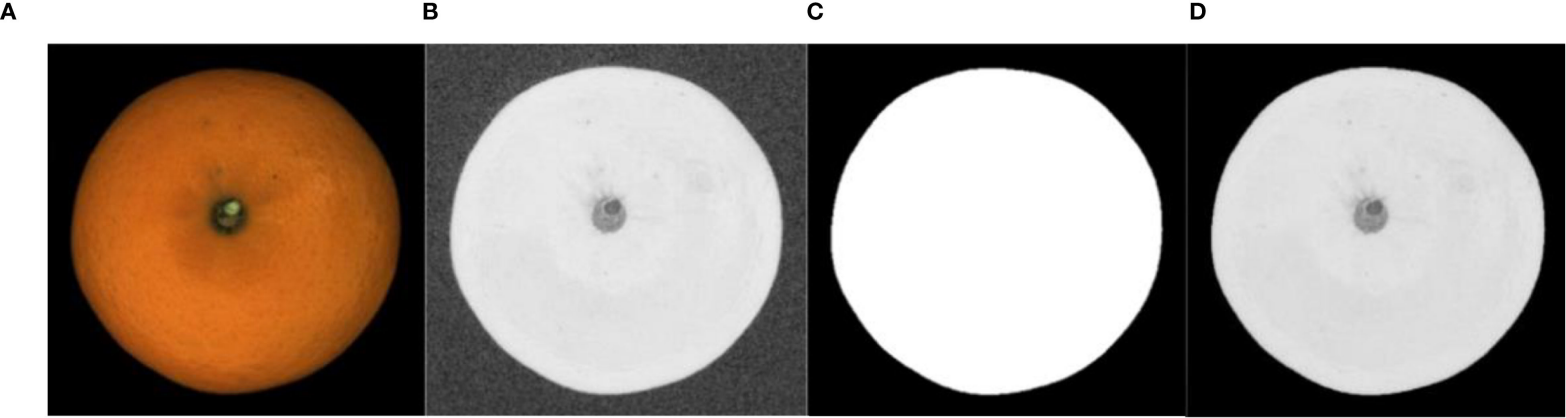
Image background segmentation. **(A)** HSV conversion, **(B)** S-channel thresholding, **(C)** Morphological refinement, **(D)** Background removal.

Experimental results demonstrate that threshold segmentation using the S component in the HSV color space yields the smoothest edges and optimal segmentation performance. As shown in **Figure 7**, when applying the binary mask in (**Figure 7B**), threshold segmentation alone fails to achieve the refined segmentation shown in (**Figure 7C**) due to interference from the fruit stem. The algorithm was particularly effective at detecting point-like defects such as thrips scarring and canker, which have clear boundaries and contrast from the healthy fruit surface. However, when applied to blocky rot defects like sunburn or rough peel, the segmentation performance was slightly reduced due to the larger, more diffuse nature of these defects. To address this, morphological operations with larger structural elements are employed to eliminate internal noise and fill holes caused by the stem (Jamil et al., 2008). The final mask (**Figure 7C**) is then used to remove the background via a bitwise AND operation with the original Orah mandarin image.

### 4.3 Sliding window algorithm for defect detection

When the sliding window detection regions are not subjected to classification processing and the maximum inter-class variance of the window regions is directly calculated as the threshold segmentation criterion, the segmentation results are shown in **Figure 2**. The algorithm successfully identified point-like defects such as canker (**Figure 2**) with high accuracy due to the sharp contrast between the defect and healthy fruit regions. In contrast, for blocky rot defects, such as sunburn (**Figure 2**), the algorithm showed slight degradation in accuracy, particularly in regions where the defect blended more uniformly with the surrounding fruit surface. During the first threshold segmentation, as illustrated in (**Figure 2A**), the numerical annotations within the window subblocks indicate the segmentation parameters calculated during sliding window processing. T represents the optimal threshold computed using the maximum inter-class variance method (Otsu’s method), and d denotes the average grayscale difference between foreground and background within the subblock. At the positions of the 4th row/2nd column and 4th row/4th column in the sliding window, it is observed that the threshold T detected by Otsu’s method is 0, resulting in failed threshold segmentation.

During the second segmentation (**Figure 2C**), when the window slides to the 3rd row/1st column and 4th row/1st column positions, threshold segmentation again fails to achieve the expected results. Specifically, significant defects are present in these regions but remain unsegmented. Analysis reveals that although relatively large inter-class grayscale differences (**Figure 2D**) are detected in these areas, the optimal threshold T remains 0. This occurs because the Orah mandarin images are preprocessed with background removal, where the detected “0” corresponds to the background value. Consequently, Otsu’s method can only segment the Orah mandarin from the background but fails to isolate defects within the Orah mandarin itself.

After presenting the sliding window defect detection results, we further analyzed the impact of morphological operations, particularly erosion, on defect retention for varying defect sizes using different structural element sizes. The results indicate that small defects, such as rust mite spots, are best preserved when smaller structural elements (e.g., 3x3 or 5x5 pixels) are used. These smaller elements allow for finer adjustments, preventing excessive erosion and preserving small, localized defects. However, this can also lead to an increase in false positives, as noise and background regions may not be effectively filtered out, leading to the misclassification of healthy regions as defects. In contrast, medium-sized defects, such as small mold spots or localized rot, benefit from medium-sized structural elements (e.g., 7x7 or 9x9  pixels), which strike a balance between removing background noise and preserving defect details. Larger defects, such as sunburn or rough peel, are more effectively detected using larger structural elements (e.g., 11x11 pixels or greater), as they help efficiently remove noise and capture broader defect areas. However, the drawback of using larger elements is that they can lead to excessive erosion, especially for defects with less distinct boundaries or those overlapping with the fruit’s surface, which results in a loss of fine details. This analysis underscores the trade-off between defect size and retention: smaller elements are ideal for small defects but may increase computational time and false positives, while larger elements are more efficient but may erode fine defect details. Balancing defect retention and computational efficiency is critical for optimizing the defect detection algorithm. Choosing the appropriate structural element size is essential for achieving optimal performance in practical applications.

As shown in **Figure 3**, (**Figure 3A**) displays the sliding window at the 4th row, 2nd column from the first segmentation pass, while (**Figure 3B**) presents the histogram statistics of the window’s pixel intensities. Theoretically, a bimodal histogram (as observed here) justifies using a threshold T=0 for binarization. However, in this study, the majority of sliding windows—particularly those at Orah mandarin edges—fall into this category. If all such windows are segmented with T=0 , numerous edge defects remain undetected. This limitation is evident in **Figure 2**, where an overwhelming number of windows yield T=0 , rendering Otsu’s method ineffective for defect segmentation (see **Figure 2**: Otsu-based threshold segmentation results).

Through analysis of the sliding window histograms, the solution for windows containing edge defects is to exclude pixels with the Orah background value (0) before calculating the threshold. Therefore, the proposed algorithm classifies the sliding window detection regions and applies distinct image processing based on predefined conditions of the detection windows. To evaluate the impact of classification processing on the sliding window algorithm, a 100 × 100-pixel sliding window was used for validation. The average inter-class grayscale difference was set to Δd = 30, and the positional offset for both the first and second image subdivisions was defined as half the window size (50 pixels). Defect segmentation was performed using two iterations of the sliding window algorithm.

**Figure 4** illustrates the first defect segmentation process using the 100 × 100-pixel sliding window: **Figure 4A** shows the initial position of the sliding window, with the scan starting from the top-left corner of the image; **Figure 4B** indicates that the sliding step size equals the full window dimension (100 pixels); **Figure 4E** demonstrates a row-wise scanning pattern, where the window shifts by its full height to the next row after completing a horizontal scan.

During defect segmentation, only the windows at the 2nd row/3rd column (**Figure 4G**), 3rd row/1st column (**Figure 4I**), and 3rd row/3rd column (**Figure 4K**) underwent threshold segmentation using the classification algorithm. All other windows directly adopted binary masks as segmentation results to reduce computational time and enhance detection efficiency.

As illustrated in **Figure 5**, the second defect detection pass employs a 100 × 100 pixel sliding window with horizontal and vertical offsets of half the window size (50 pixels) relative to the origin (top-left corner of the image). During this pass, threshold segmentation is selectively applied only to: 2nd row, 2nd column (**Figure 5E**), 3rd row, 1st column (**Figure 5G**), and Other sub-windows retain their prior segmentation results without additional thresholding operations.

**Figure 8:**
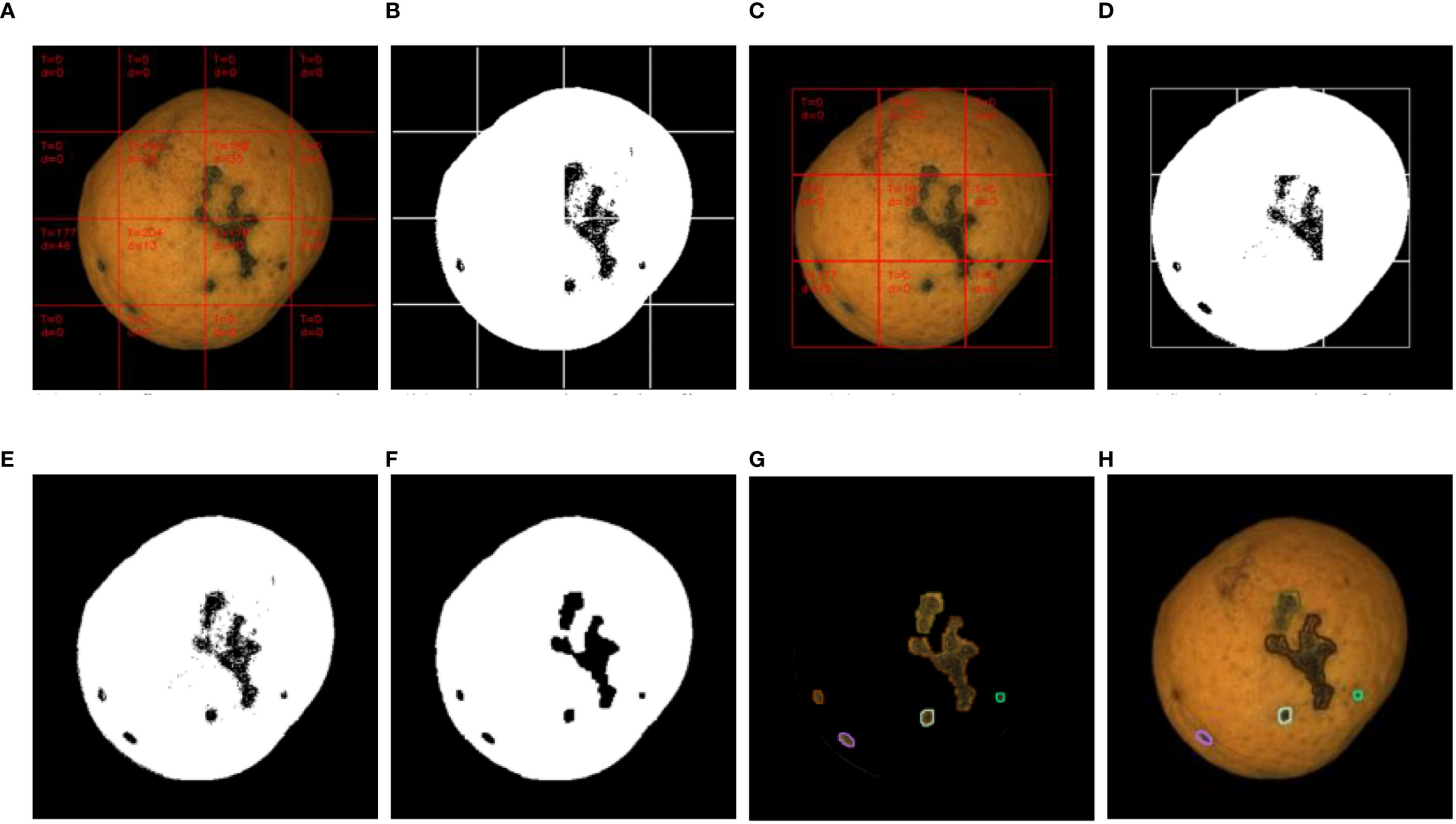
Sliding window defect detection process. **(A)** Original Orah mandarin image with surface defects, **(B)** Binary mask after initial threshold segmentation, **(C)** Defect map after morphological refinement, **(D)** Sliding window positioning grid, **(E)** Inter-class grayscale difference visualization, **(F)** Fused defect detection result, **(G)** Final defect segmentation with contour overlay, **(H)** Defect localization on color image.

As shown in **Figure 8**, the visualization process of defect detection using the 100 × 100-pixel sliding window algorithm is demonstrated. (**Figures 8A, C**) display the optimal segmentation thresholds and inter-class average grayscale differences calculated for each sub-block during the first and second threshold segmentation passes, respectively, based on the proposed algorithm. The values within each sub-block reveal that only 5 localized threshold binarization calculations were performed across the entire Orah mandarin target image through two sliding window iterations, completing defect extraction. In contrast, 25 threshold segmentation operations would have been required without the three-category classification of sliding window regions. This optimized algorithm significantly enhances processing speed for defect detection. (**Figure 8E**) illustrates the fused defect detection result from both passes, which initially contained numerous holes. Morphological operations were therefore applied to fill these voids and eliminate burrs. The final refined defect detection result (**Figure 8G**) is presented with color-coded contours superimposed on the target image (**Figure 8H**) for enhanced visual clarity.

**Figure 9:**
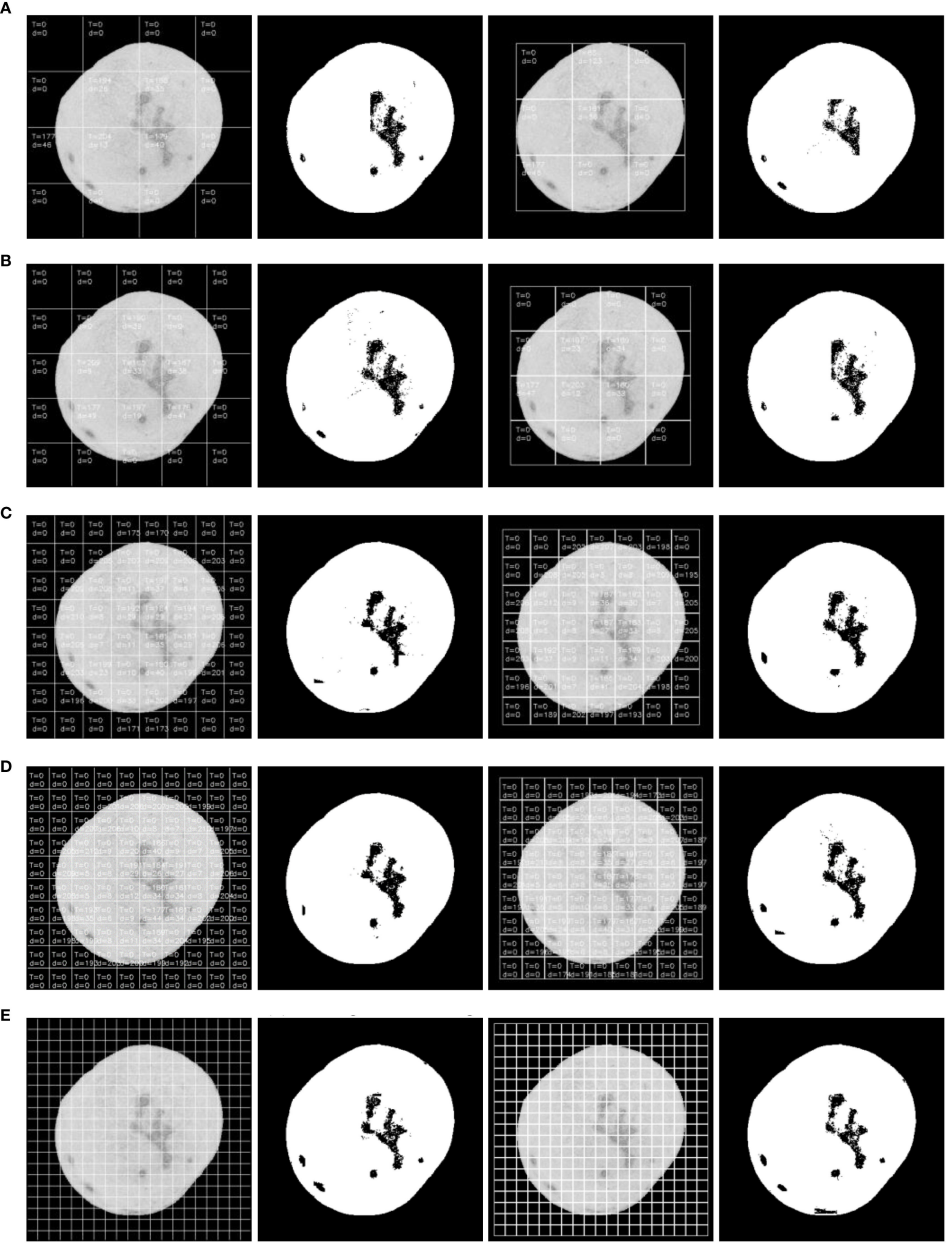
Segmentation effect of sliding window with different sizes. **(A)** 100×100 pixels, **(B)** 80×80 pixels, **(C)** 50×50 pixels, **(D)** 40×40 pixels, **(E)** 20×20 pixels.

The core of the sliding window threshold segmentation algorithm proposed in this paper lies in the configuration of the window size and the selection of the average inter-class grayscale difference ( Δd ). To validate the algorithm’s effectiveness and optimize parameters, multiple sliding window sizes were tested using a classification algorithm for threshold segmentation on detection regions. As shown in **Figure 9**, defect segmentation results vary significantly with different window dimensions. Analysis reveals that smaller windows (e.g., 20 × 20 pixels) achieve higher precision, enabling complete defect segmentation in a single scan. This improvement stems from the enhanced uniformity of illumination and stronger pixel correlations within smaller regions, which simplify foreground-background differentiation. However, smaller windows exponentially increase computational overhead due to the larger number of subregions requiring processing. When using a 100 × 100-pixel window (see **Figure 9A**), the first scan detects most prominent defects but misses subtle ones. This occurs because subregions with defect pixels below the classification algorithm’s threshold are replaced with binary masks, bypassing segmentation. Additionally, the large window size leads to non-uniform brightness distribution, particularly near Orah edges, further reducing detection reliability. To address these limitations, the algorithm incorporates a second detection pass with redefined subblocks. New subblocks cover 1/4 of the original area, combining pixels from the original subblock and three adjacent neighbors. This reconfiguration alters pixel correlations and expands detection scope. As shown in **Figure 9**’s third column, subblocks are offset by half the window size (e.g., 50 pixels for 100 × 100 windows), reducing the total number of windows while revisiting overlooked regions. Although some defects detected in the first pass may be missed due to positional shifts, the fusion of results from both scans yields a comprehensive defect map. In summary, while smaller windows enhance precision, they demand greater computational resources. Larger windows prioritize efficiency but sacrifice accuracy. The two-stage detection strategy balances these trade-offs, leveraging subblock redefinition and positional offsets to optimize defect identification in practical applications.

As shown in **Table 2**, the statistical comparison of processing time and accuracy for defect detection using different sliding window sizes reveals significant trends. During the first threshold segmentation pass, the 100 × 100-pixel window required only 56.7 milliseconds, while the 20 × 20-pixel window took approximately 260 milliseconds—a fivefold increase in time. The gap widened further during the second pass, reaching a sevenfold difference. This discrepancy likely arises because smaller windows exhibit stronger pixel correlations and more uniform light intensity distributions within their regions, facilitating easier defect segmentation. However, this advantage comes at the cost of exponentially increasing the number of windows requiring processing, thereby prolonging computation time. The even greater time consumption during the second pass suggests that the positional offset of the sliding windows enhances pixel correlation within the new detection regions, improving coverage of defect areas and boosting detection precision. The recognition accuracy of the 20 × 20-pixel window shows a marked improvement over the 100 × 100-pixel window, as evidenced by the detection rates. This confirms that smaller window sizes enhance precision but at the expense of substantially increased computational time. Consequently, practical applications must balance recognition accuracy and processing efficiency by selecting an appropriate window size tailored to specific requirements. In addition to processing time, the performance of the defect detection algorithm is assessed using accuracy, IoU, and Dice. These metrics provide a more comprehensive evaluation of the algorithm’s performance:

**Table 2:** Time consuming and recognition accuracy of sliding windows of different sizes.

Sliding window sizes	The first segmentation time (ms)	The second segmentation time (ms)	Total segmentation time (ms)	Recognition accuracy (%)	IoU (%)	Dice (%)
100×100	56.7	25.6	85.3	97.5	92.81	96.27
80×80	57.7	35.1	92.8	97.8	92.84	96.29
50×50	87.3	49.8	137.1	98.3	93.28	96.52
40×40	108.5	79.2	187.7	98.4	93.54	96.66
20×20	260.5	192.9	453.4	98.9	93.41	96.60

Recognition accuracy: The ratio of correctly detected defects to the total number of defects, defined as in Equation (9).


(9) $$\text{Recognition accuracy}=\frac{\text{TP}}{\text{TP}+\text{FN}}\times 100\%,$$

where TP represents true positives and FN represents false negatives.

Intersection over Union (IoU): The ratio of the intersection and union of the predicted and ground truth defect regions, calculated as in Equation (10).


(10) $$\text{IoU}=\frac{\text{TP}}{\text{TP}+\text{FP}+\text{FN}}\times 100\%,$$

where FP represents false positives.

Dice Coefficient (Dice): A measure of the similarity between the predicted and ground truth regions, calculated as in Equation (11).


(11) $$\text{Dice}=\frac{2\times \text{TP}}{2\times \text{TP}+\text{FP}+\text{FN}}\times 100\%,$$

As window sizes decrease, both IoU and Dice show an improvement, indicating higher precision in detecting defects, particularly for smaller and more challenging regions. These improvements in spatial overlap and detection accuracy validate the effectiveness of smaller window sizes for defect detection, although they come with the trade-off of increased computational time.

To evaluate the effectiveness of sliding window overlap, a secondary experiment was conducted with a 50% overlap between consecutive sliding windows. In this modified strategy, the sliding window step size was adjusted to d_step=n/2, where *n* is the window size. This adjustment allows the detection of defects that may straddle the boundary of two adjacent windows in the traditional non-overlapping approach. Experimental results indicated that the 50% overlap enhanced the detection of smaller or partial defects that might have been missed with the original approach, leading to a more accurate overall defect recognition rate.

### 4.4 Stem detection

**Figure 10:**
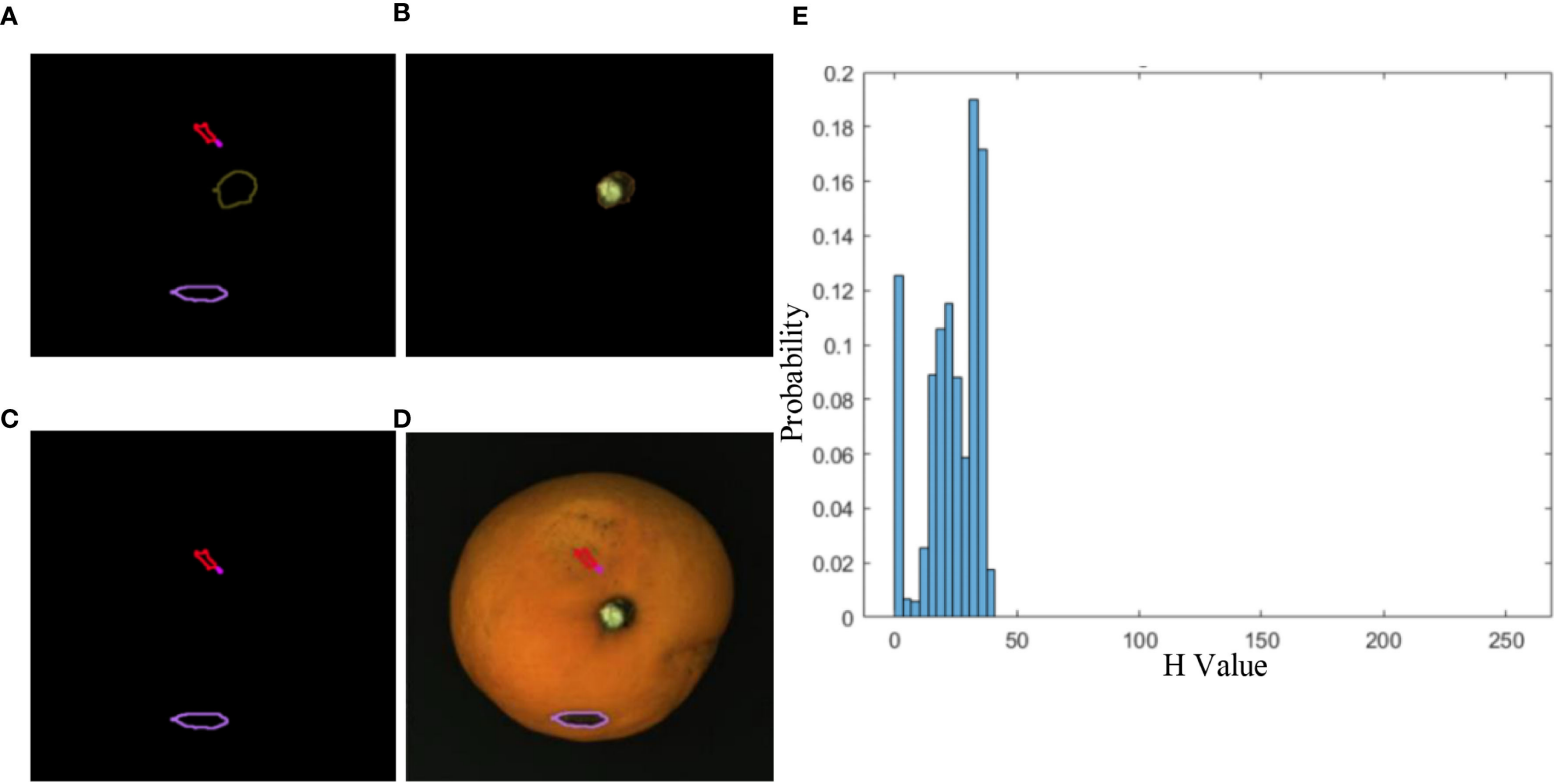
The circularity and hue analysis for stem detection. **(A)** Initial defect markers, **(B)** Stem region extraction, **(C)** Circularity analysis, **(D)** Hue spatial mapping, **(E)** Hue distribution histogram.

The fruit stem typically exhibits a different hue compared to the Orah mandarin and has an approximately elliptical shape. This paper proposes a method combining circularity and hue analysis for stem detection, as illustrated in **Figure 10**. After defect detection using a sliding window, the marked defects are shown in (**Figure 10A**). The fruit stem regions extracted by our detection algorithm are displayed in (**Figure 10B**), with their corresponding hue channel histogram presented in (**Figure 10E**). The histogram reveals that the hue values of stem regions predominantly fall within the 23–35 degree range. In this study, defect regions with a circularity score exceeding 0.8 and an average hue value between 23–35 degrees are identified as fruit stems. Testing on collected experimental samples demonstrated a detection accuracy of 96%. The algorithm features relative simplicity, ease of implementation and deployment, along with high computational efficiency, making it suitable for real-time or rapid stem detection scenarios.

### 4.5 Light robustness test

To evaluate the robustness of the defect detection algorithm under different lighting conditions, we added two experimental scenarios: simulated overcast (240 lux) and strong reflection (500 lux). In the simulated overcast scenario, the lighting was weak, resulting in lower image contrast, which could affect defect recognition. In the strong reflection scenario, excessive lighting or reflections caused glare and overexposure, which could hinder defect detection. The experimental results showed that the algorithm performed well under the simulated overcast condition, maintaining a high detection accuracy, demonstrating its robustness in low-light environments. However, in the strong reflection scenario, detection accuracy slightly decreased, but morphological refinement steps effectively removed some noise, ensuring valid defect detection. Overall, the algorithm showed stable performance under both lighting conditions, particularly in low-light environments, where it demonstrated strong adaptability. While performance slightly decreased under high-reflection conditions, it was still able to cope with strong lighting effects. Future work can further optimize the algorithm to improve performance under extreme lighting conditions.

To assess the algorithm’s performance under these lighting scenarios. We calculated the False Positive Rate (FPR) and False Negative Rate (FNR) for each experimental setup, as shown in Equations 12, 13, and the results under different lighting conditions are summarized in **Table 3**.


(12) $$\text{FPR}=\frac{\text{FP}}{\text{FP}+\text{TN}},$$

(13) $$\text{FNR}=\frac{\text{FN}}{\text{FN}+\text{TP}},$$

**Table 3:** Performance of sliding window algorithm under different lighting.

Lighting condition	Detection accuracy (%)	False positive rate(%)	False negative rate(%)	Average processing time (ms)
Simulated Overcast (240 lux)	56.7	25.6	85.3	97.5
Strong Reflection (500 lux)	57.7	35.1	92.8	97.8

Where FP (False Positives) refers to the number of normal regions incorrectly classified as defects, TN (True Negatives) represents the number of normal regions correctly classified as normal, FN (False Negatives) is the number of defect regions that the algorithm failed to detect, and TP (True Positives) indicates the number of defect regions correctly identified as defects.

### 4.6 Ablation study

To further investigate the impact of key parameters on the defect detection algorithm, we conducted an ablation study focusing on two critical parameters: Δd  (inter-class grayscale difference threshold) and circularity threshold. We tested different values of these parameters and analyzed their effects on detection accuracy, false positive rates, and computational efficiency. First, we evaluated the effect of different  Δd  values (e.g., 20, 30, and 40) on detection accuracy and computational time. As shown in **Table 4**, smaller  Δd  values (e.g., 20) improved the detection accuracy of small defects but also resulted in a higher false positive rate, especially in regions where the grayscale difference between the foreground and background was small. Larger Δd values (e.g., 40) reduced false positives but missed smaller defects, leading to a reduction in detection accuracy. Δd = 30  provided the best balance, achieving good performance in both detection accuracy and computational efficiency.

**Table 4:** Impact of Δd on performance.

Δd	Segmentation time (ms)	Detection accuracy (%)	False positive rate(%)
20	75.6	96.2	4.3
30	85.3	97.5	2.0
40	95.8	95.8	1.5

In addition to Δd , the sliding window size plays a crucial role in determining the effectiveness of the defect detection algorithm. Smaller sliding windows (e.g., 20×20 pixels) provide finer granularity, making them more capable of detecting small defects. However, this comes at the cost of increased computation time due to the larger number of regions to process as shown in **Table 5**. In contrast, larger windows (e.g., 100×100 pixels) reduce the computation time by processing fewer windows but may miss smaller or subtle defects, particularly at the edges of the fruit. Based on our experiments, we found that a sliding window size of 100×100 pixels provided an optimal balance between detection accuracy and computational speed for the majority of defect types encountered in Orah mandarin. This choice ensured that the algorithm could detect both large and small defects without significantly increasing the processing time.

**Table 5:** Time consuming and recognition accuracy of sliding windows with different sizes and overlap strategies.

Sliding window sizes	$d_{step}$	The first segmentation time (ms)	The second segmentation time (ms)	Total segmentation time (ms)	Recognition accuracy (%)	IoU (%)	Dice (%)
100×100	n	56.7	25.6	85.3	97.5	92.81	96.27
100×100	$\frac{n}{2}$	57.7	35.1	92.8	97.8	92.84	96.29
50×50	n	87.3	49.8	137.1	98.3	93.28	96.52
50×50	$\frac{n}{2}$	108.5	79.2	187.7	98.4	93.54	96.66

Next, we analyzed the effect of circularity threshold (set at 0.7, 0.8, and 0.9) on stem detection accuracy and false positive rates. As shown in **Table 6**, lower circularity thresholds (e.g., 0.7) resulted in a higher false positive rate as non-stem regions were misclassified as stems. Higher thresholds (e.g., 0.9) reduced false positives but led to lower detection accuracy, as some irregularly shaped stems were missed.  Circularity threshold = 0.8 provided the best balance, offering high accuracy and low false positive rates.

**Table 6:** Impact of circularity threshold on performance: the bold values indicate optimal threshold.

Circularity threshold	Segmentation time (ms)	Detection accuracy (%)	False positive rate(%)
0.7	80.5	96.1	3.8
**0.8**	**85.3**	**97.5**	**2.0**
0.9	90.2	95.2	1.2

By comparing **Table 4** and **Table 6**, it is evident that Δd = 30 and circularity threshold = 0.8 provided the best detection accuracy and the lowest false positive rate. These experimental results highlight the importance of selecting the right parameters for optimal performance. The combination of these two parameters ensures the best balance between detection precision and computational efficiency.

### 4.7 Method comparison

To evaluate the effectiveness of the proposed method, we conducted a comparative analysis with the traditional Retinex method and U-Net model. Both methods are more appropriate for comparison with the proposed method due to their focus on pixel-level segmentation. The comparison metrics include recognition accuracy, computation time, false positive rate, and intersection over union (IoU).

As shown in **Table 7**, the proposed method outperforms both Retinex and U-Net in recognition accuracy (97.5%) and false positive rate (2.0%), while also achieving the fastest computation time (85.3 ms per fruit). In contrast, The Retinex is computationally expensive, requiring 152.7 ms per fruit, making it unsuitable for real-time applications. The U-Net requires 120.5 ms per fruit, which still demands substantial computational resources. While U-Net achieves the Dice coefficient (93.50%) and IoU (90.45%), it is less efficient in processing time and requires more resources. The proposed method, however, achieves competitive IoU (92.81%) and Dice (96.27%) scores, offering a better balance between detection accuracy and computational efficiency.

**Table 7:** Comparison of the proposed method with retinex and U-Net: the bold values indicating best performance.

Method	Recognition accuracy(%)	Computation time(ms)	False positive rate (%)	IoU (%)	Dice (%)
Proposed Method	**97.5**	**85.3**	**2.0**	**92.81**	**96.27**
Retinex	94.5	152.7	4.5	89.78	93.40
U-Net	95.8	120.5	3.5	90. 45	93.50

## 5 Conclusion

To address the shortcomings of traditional illumination correction algorithms—such as high computational cost, time-consuming processes, and elevated false defect detection rates—this study proposes an external defect detection algorithm for Orah mandarin (a citrus cultivar) that eliminates the need for brightness correction. The algorithm first divides the captured Orah mandarin image into multiple uniformly sized regions and performs threshold segmentation on each region sequentially using a sliding window. Next, the threshold-segmented regions are merged while removing fruit stem areas. Finally, morphological operations are applied to eliminate noise and obtain complete defect segmentation results. To validate the algorithm’s performance, 100 Orah mandarin images with diverse defect features were collected in real-world settings and tested using the proposed method. Experimental results show that with a sliding window size of 100×100 pixels, the algorithm achieves an external defect detection speed of 85.3 ms per fruit and a defect recognition rate of 97.5%. When using a 20×20 pixel sliding window, the defect recognition rate increases to 98.9%. Additionally, the fruit stem detection accuracy reaches 96%. The proposed algorithm effectively resolves the issue of low detection accuracy caused by uneven surface illumination distribution on spherical fruits, significantly improving detection speed by avoiding complex brightness correction processes. Future work will explore the use of parallel computing technologies to further enhance processing efficiency and scalability.

The choice of sliding window size and circularity threshold was determined through comprehensive sensitivity analysis. Smaller sliding window sizes (e.g., 20×20 pixels) were found to improve detection accuracy, particularly for smaller defects, but this came at the cost of significantly increased computational time due to the larger number of windows processed. In contrast, larger window sizes (e.g., 100×100 pixels) enhanced processing speed but risked missing smaller defects, especially those located at the fruit’s edges. Through a balance of computational efficiency and detection accuracy, we selected the 100×100 pixel sliding window as optimal, which was validated through ablation experiments. Similarly, the circularity threshold, set at 0.8, provided the best balance between detection accuracy and reducing false positives. A lower circularity threshold (e.g., 0.7) resulted in higher false positive rates, while a higher threshold (e.g., 0.9) reduced false positives but led to the omission of some irregularly shaped stems. The 0.8 threshold was optimal for maintaining accuracy while minimizing misclassifications.

Compared to traditional Histogram Equalization and Retinex algorithms, the proposed method offers notable advantages. Both Histogram Equalization and Retinex are effective in correcting uneven lighting, but they come with high computational costs and the potential for over-enhancement, which can distort critical image details. These methods are often slow, making them less suitable for real-time applications in large datasets or low-resource environments. In contrast, our algorithm avoids these preprocessing stages, providing faster defect detection while maintaining high accuracy, especially in handling the uneven illumination of spherical fruits like Orah mandarins.

Additionally, while deep learning-based methods like YOLOv8 excel in defect detection accuracy, they come with the drawback of substantial computational overhead, requiring significant hardware resources for training and inference. YOLOv8, although offering high precision, may not be as efficient in time-sensitive or resource-constrained applications. In comparison, our approach strikes a better balance between speed and accuracy, achieving real-time detection without the need for extensive computational resources.

The proposed algorithm effectively resolves the issue of low detection accuracy caused by uneven surface illumination distribution on spherical fruits, while significantly improving detection speed by avoiding complex brightness correction processes. Future work will explore the use of parallel computing technologies to further enhance the algorithm’s speed and deploy it on embedded devices for real-time online detection of Orah mandarin external defects.

## Data Availability

The original contributions presented in the study are included in the article/supplementary material. Further inquiries can be directed to the corresponding author.
